# A Multi-Phase Analytical Model for Effective Electrical Conductivity of Polymer Matrix Composites Containing Micro-SiC Whiskers and Nano-Carbon Black Hybrids

**DOI:** 10.3390/polym17020128

**Published:** 2025-01-07

**Authors:** Usama Umer, Mustufa Haider Abidi, Zeyad Almutairi, Mohamed K. Aboudaif

**Affiliations:** 1 Advanced Manufacturing Institute, King Saud University, P.O. Box 800, Riyadh 11421, Saudi Arabia; 2 Mechanical Engineering Department, College of Engineering, King Saud University, P.O. Box 800, Riyadh 11421, Saudi Arabia

**Keywords:** electrical property, polymer composite, hybrid fillers, aspect ratio, multi-phase model

## Abstract

Multifunctional polymer composites containing micro/nano hybrid reinforcements have attracted intensive attention in the field of materials science and engineering. This paper develops a multi-phase analytical model for investigating the effective electrical conductivity of micro-silicon carbide (SiC) whisker/nano-carbon black (CB) polymer composites. First, CB nanoparticles are dispersed within the non-conducting epoxy to achieve a conductive CB-filled nanocomposite and its electrical conductivity is predicted. Some critical microstructures such as volume percentage and size of nanoparticles, and interphase characteristics surrounding the CB are micromechanically captured. Next, the electrical conductivity of randomly oriented SiC-containing composites in which the nanocomposite and whisker are considered as the matrix and reinforcement phases, respectively, is estimated. Influences of whisker aspect ratio and volume fraction on the effective electrical conductivity of the SiC/CB-containing polymer composites are explored. Some comparison studies are performed to validate the accuracy of the model. It is observed before the percolation threshold that the addition of nanoparticles with a uniform dispersion can improve the electrical conductivity of the polymer composites containing SiC/CB hybrids. Moreover, the results show that the electrical conductivity is more enhanced by the decrease in nanoparticle size. Interestingly, the composite percolation threshold is significantly reduced when SiC whiskers with a higher aspect ratio are added. This work will be favorable for the design of electro-conductive polymer composites with high performances.

## 1. Introduction

Polymer composites containing micro/nano hybrid reinforcements have increasingly attracted attention in materials science, particularly in applications requiring both lightweight and high electrical conductivity [1,2]. Polymer composite materials, despite their widespread use in structural applications, suffer from inherently low electrical conductivity, particularly in non-conductive polymer matrices ($\sim 10^{-15}–10^{-9}\;S/m$), which limits their functionality in aerospace and electronics industries [1,2]. This necessitates the addition of conductive nanofillers to significantly enhance electrical conductivity while retaining the mechanical advantages of the composite structure [3,4].

Silicon carbide whiskers and carbon black nanoparticles are emerging as effective hybrid fillers due to their potential to enhance electrical properties, making them ideal for advanced applications in electronics, energy storage, and aerospace. Zhang et al. fabricated a composite with copper matrix and SiC whiskers as the reinforcing phase by means of the co-electrodeposition technology [5]. They concluded that the manufactured composite displays improved shear strength and enhanced thermal conductivity. Fan and colleagues presented a comprehensive review of the application of carbon black as the reinforcing phase [6]. First, the physical and chemical characteristics of CB were characterized in conjunction with the mixing procedure, and afterward, eight reinforcing mechanisms of CB were listed and summarized. The inclusion of CB nanoparticles into a non-conductive polymer matrix can transform the material into a conductive composite, primarily through the formation of conductive networks at the percolation threshold [7,8]. However, a critical challenge with CB is that achieving percolation often requires a high volume fraction of CB, which can compromise the mechanical integrity of the composite [9]. Mazaheri et al. scrutinized the electrical conductivity and percolation characteristics of polymer nanocomposites (PNCs) consisting of CB spherical particles as the reinforcing phase [10]. Their analytical modeling considered the influence of interphase region, quantum electron tunneling, volume fraction, radius, and filler/interphase/matrix conductivities. Despite the high percolation threshold, CB remains a popular choice due to its cost-effectiveness and superior electrical properties compared to other nanofillers such as carbon nanotubes (CNTs) and graphene nanoplatelets (GNPs). CB exhibits a rapid increase in electrical conductivity near the percolation point, primarily driven by electron tunneling between particles [11,12]. However, the need for large amounts of CB can result in filler aggregation and reduced processability, limiting its performance in polymer nanocomposites [13,14].

To address these limitations, hybrid systems combining micro- and nano-sized fillers have been explored to optimize electrical performance while reducing filler content. Kim et al. proposed an analytical method for composites containing multiple heterogeneities with conductive coated layers to estimate the percolation threshold influence, the tunneling influence by hard/soft core concept as well as the electrical conductivity of polymeric composites with ellipsoidal fillers [12]. Saberi et al. explored the electrical conductivity of short carbon fiber-reinforced nanocomposites enhanced with GNPs [15]. To this end, a two-phase micromechanical modeling method was used to predict the effective electrical conductivity of composites. They concluded that the hybrid nanocomposite displayed better conductivity by increasing the volume fraction and aspect ratio of the GNP and reducing its thickness. They also deduced that increasing the carbon fiber aspect ratio results in higher conductivity. Previously, an analytical modeling approach was proposed to investigate the electrical conductivity of hybrid CNT/CB/polymer nanocomposites [16]. The model incorporated cylindrical nanotubes and spherical CB fillers covered by an interphase region serving as an electron hopping duct. Various materials may be used as the reinforcing phase to enhance the electrical conductivity of polymeric composites which require further investigation. In particular, SiC whiskers, known for their high aspect ratio, mechanical strength, and good electrical properties, may provide a complementary scale to CB, enhancing the formation of multi-scale conductive networks [17]. This synergistic interaction between SiC whiskers and CB nanoparticles may significantly lower the percolation threshold while improving electrical conductivity, as the whiskers serve as bridges within the composite, facilitating more efficient electron transport [18,19,20].

In this work, an analytical micromechanics method with some important microstructures for predicting the effective electrical conductivity of polymer composites reinforced with micro-SiC whiskers and CB nanoparticles is proposed which, to date, has not been studied. This model captures critical microstructural parameters, including the volume fraction and size of the CB nanoparticles, the interphase characteristics, and the aspect ratio of the SiC whiskers. Initially, the electrical conductivity of the CB-filled nanocomposite is predicted, followed by an analysis of the hybrid SiC/CB system, where the SiC whiskers act as reinforcement within the conductive nanocomposite matrix. The percolation behavior and the influence of whisker geometry and content are explored using a micromechanics-based approach.

## 2. Analytical Modeling

**Figure 1:**
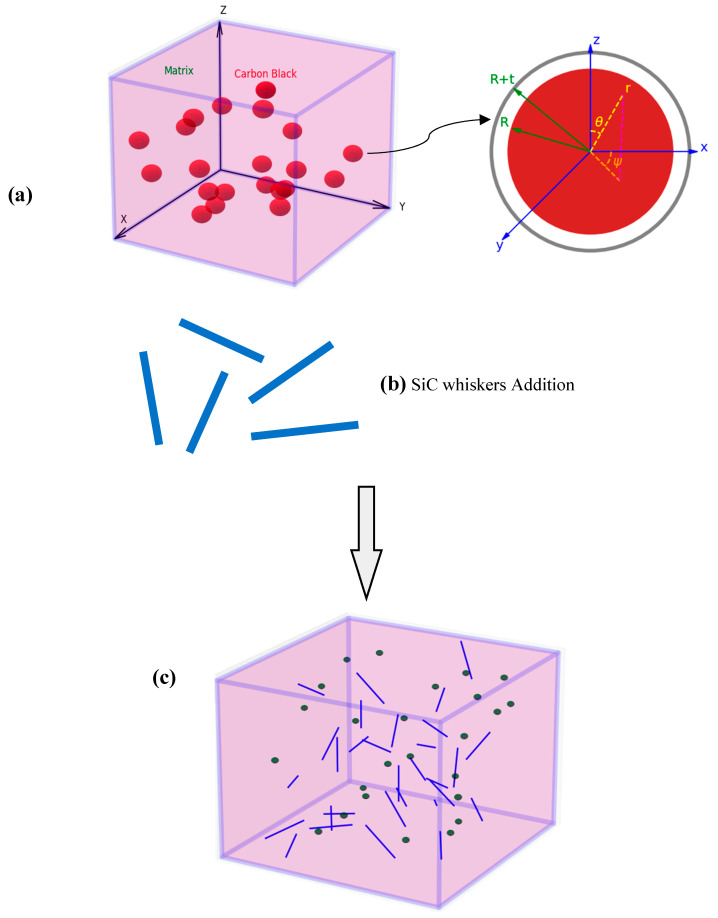
(**a**) RVE representing the random dispersion of CB nanoparticles, (**b**) SiC whiskers and (**c**) RVE for the CB/SiC/polymer ternary hybrid composite.

The analytical procedure to estimate the equivalent electrical conductivity of the polymer composite containing SiC/CB hybrids starts with the estimation of the electrical conductivity of the polymeric nanocomposite material containing CB nanoparticles a priori. For this purpose, incorporating the interaction between the nanoparticles and the polymer may be a significant issue [12,13,14,15,16]. An equivalent solid continuum interphase region is captured among the carbon-based nanoparticles and the polymer characterizing the interaction. Subsequently, incorporating the polymeric nanocomposite as the matrix phase and the SiC whisker as the reinforcements, the electrical conductivity of the hybrid composite can be calculated. Figure 1a presents a schematic representation of the initial configuration of the composite, depicted within a global XYZ coordinate system. The figure illustrates the dispersion of CB nanoparticles, represented as red spheres, within a non-conducting polymer matrix, shown in a purple hue. The coordinate axes are labeled with the X, Y, and Z directions indicating the spatial arrangement of the representative volume element (RVE).

Additionally, the inset diagram highlights the geometric parameters associated with the CB particles, including their radius *R* and the thickness of the surrounding interphase *t*, which collectively contribute to the effective electrical properties of the composite. Figure 1b shows the addition of SiC whiskers. Subsequently, Figure 1c illustrates the final configuration of the hybrid composite, where the SiC whiskers, represented as blue lines, are randomly oriented and dispersed throughout the CB-filled polymer matrix. This arrangement is crucial for understanding the interactions between the micro and nano reinforcements and their collective impact on the electrical conductivity of the hybrid composite. The overarching goal of this representation is to facilitate a comprehensive analysis of the structural and electrical characteristics of the CB/SiC hybrid composite, ultimately leading to enhanced performance in electro-conductive applications.

### 2.1. Effective Electrical Conductivity of CB-Reinforced Nanocomposites

The initial step to determine the electrical conductivity of the polymeric nanocomposite depicted in Figure 1a is the calculation of the electrical conductivity of the effective CB nanofiller consisted of CB nanoparticle and interphase region. Then, the integration of CB nanofiller with the polymer matrix is taken into account accordingly.

#### 2.1.1. Electrical Conductivity of Effective CB Nanofillers

The electrical potential and conductivity of effective spherical CB nanoparticles under an applied electric field can be derived by solving Laplace’s equation in spherical coordinates with appropriate boundary conditions. The equation governing the electric potential Φ in spherical coordinates is given as follows [10,16,21]:

(1) $$\nabla^{2}\Phi =\frac{1}{r^{2}}\frac{\partial }{\partial r}\left(r^{2}\frac{\partial \Phi }{\partial r}\right)+\frac{1}{r^{2}sin\theta }\frac{\partial }{\partial \theta }\left(sin\theta \frac{\partial \Phi }{\partial \theta }\right)=0$$

where Φ depends on the radial distance r and the polar angle θ. To solve for the electric potential, boundary conditions are applied at the interfaces between the CB core, the surrounding interphase layer, and the polymer matrix. Denoting the electric potentials in the CB core, the interphase, and the matrix as $\Phi_{CB}$, $\Phi_{int}$, and $\Phi_{m}$, respectively, the boundary conditions at R and R+t are as follows [10,16]:

(2) $$\Phi_{CB}{|}_{r=R}=\Phi_{int}{|}_{r=R}$$

(3) $$-\sigma_{CB}\frac{\partial \Phi_{CB}}{\partial r}{|}_{r=R}=-\sigma_{int}\frac{\partial \Phi_{int}}{\partial r}{|}_{r=R}$$

(4) $$\Phi_{int}{|}_{r=R+t}=\Phi_{m}{|}_{r=R+t}$$

(5) $$-\sigma_{int}\frac{\partial \Phi_{CB}}{\partial r}{|}_{r=R+t}=-\sigma_{m}\frac{\partial \Phi_{int}}{\partial r}{|}_{r=R+t}$$

where $\sigma_{CB}$, $\sigma_{int}$, $\sigma_{m}$ denote the electrical conductivities of the CB core, the interphase, and the polymer matrix, sequentially. The azimuthal symmetry of the system implies that the potential solution is independent of the azimuthal angle, reducing Laplace’s equation to the following general solution for each region [10,16,22]:

(6) $$\Phi_{CB}\left(r,\theta \right)=\sum_{n=0}^{\infty }(A_{n}r^{n}+\frac{B_{n}}{r^{n+1}})P_{n}\left(cos\theta \right),\;\;r\leq R$$

(7) $$\Phi_{int}\left(r,\theta \right)=\sum_{n=0}^{\infty }(A_{n}^{\prime }r^{n}+\frac{B_{n}^{\prime }}{r^{n+1}})P_{n}\left(cos\theta \right),\;\;R\leq r\leq R+t$$

(8) $$\Phi_{m}\left(r,\theta \right)=\sum_{n=0}^{\infty }(A_{n}^{\prime \prime }r^{n}+\frac{B_{n}^{\prime \prime }}{r^{n+1}})P_{n}\left(cos\theta \right),\;\;r\geq R+t$$

By applying the boundary conditions, the coefficients are determined, and the electric potential simplifies to the first-order terms (since higher-order terms vanish). Thus, the electric potentials in the three regions become the following:

(9) $$\Phi_{CB}\left(r,\theta \right)=A_{1}rcos\theta ,\;\;r\leq R$$

(10) $$\Phi_{int}\left(r,\theta \right)={(A}_{1}^{\prime }r+\frac{B_{1}^{\prime }}{r^{2}})cos\theta ,\;\;R\leq r\leq R+t$$

(11) $$\Phi_{m}\left(r,\theta \right)=\left(-E_{0}r+\frac{B_{1}^{\prime \prime }}{r^{2}}\right)cos\theta ,\;\;r\geq R+t$$

The coefficients of the above equation are obtained from the boundary conditions as follows:

(12) $$A_{1}=\frac{-9\sigma_{int}\sigma_{m}E_{0}}{\left(\sigma_{int}{+2\sigma }_{m}\right)\left(2\sigma_{int}+\sigma_{CB}\right)-\frac{2R^{3}}{{\left(R+t\right)}^{3}}\left(\sigma_{int}{-\sigma }_{m}\right)\left(\sigma_{int}{-\sigma }_{CB}\right)}\;$$

(13) $$A_{1}^{\prime }=\frac{2\sigma_{int}+\sigma_{CB}}{3\sigma_{int}}A_{1}$$

(14) $$B_{1}^{\prime }=\frac{\sigma_{int}-\sigma_{CB}}{3\sigma_{int}}{R^{3}A}_{1}$$

(15) $$B_{1}^{\prime \prime }={\left(R+t\right)}^{3}(A_{1}^{\prime }+\frac{B_{1}^{\prime }}{{\left(R+t\right)}^{3}}+E_{0})$$

In addition, the electric fields (E=−∇Φ) within the CB core and the interphase region are as follows:

(16) $$E_{CB}^{i}=-\frac{\partial \Phi_{CB}}{\partial j}=-\frac{1}{cos\theta }\frac{\partial \Phi_{CB}}{\partial r}=-A_{1}$$

(17) $$E_{int}^{i}=-\frac{\partial \Phi_{int}}{\partial j}=-\frac{1}{cos\theta }\frac{\partial \Phi_{int}}{\partial r}={-A}_{1}^{\prime }+\frac{2B_{1}^{\prime }}{r^{3}},$$

where i∈{x, y, z}. The equivalent electrical conductivity of the spherical CB nanoparticle $\left(\sigma_{CB}\right)$ is derived by solving the current density relation $J=\sigma_{CB}E$, where J and E are the spatially averaged current densities and electric fields, in sequence. This yields the following expression for the effective conductivity of a CB nanoparticle [10,16]:

(18) $$\sigma_{CB}^{i}=\left[\frac{3\sigma_{CB}\sigma_{int}R^{3}+\sigma_{int}\left(2\sigma_{int}+\sigma_{CB}\right)\left[{\left(R+t\right)}^{3}-R^{3}\right]-6\sigma_{int}\left(\sigma_{int}-\sigma_{CB}\right)ln\left[\left(R+t\right)/R\right]}{3\sigma_{int}R^{3}+\left(2\sigma_{int}+\sigma_{CB}\right)\left[{\left(R+t\right)}^{3}-R^{3}\right]-6\left(\sigma_{int}-\sigma_{CB}\right)ln\left[\left(R+t\right)/R\right]}\right]$$

#### 2.1.2. Electrical Conductivity of CB/Polymer Nanocomposites

In a composite system where CB nanoparticles are dispersed within a polymer matrix, the electrical conductivity $\sigma_{t}$ is derived using a mean-field approximation. According to Ohm’s law, the current density $\langle J\rangle =\sigma_{t}\langle E\rangle$ relates to the external electric field $E_{0}$. The internal current densities of the CB nanoparticles $J_{CB}^{in}$ and the matrix $J_{m}^{in}$ are given as follows [10,16,23,24]:

(19) $$J_{CB}^{in}=\sum_{i=x,\;y,\;z}\sigma_{CB}^{i}\frac{\sigma_{t}}{\sigma_{t}+B_{CB}^{i}(\sigma_{CB}^{i}-\sigma_{t})}E_{0}$$

(20) $$J_{m}^{in}=\sum_{k=X,Y,Z}\sigma_{m}^{k}\frac{\sigma_{t}}{\sigma_{t}+B_{m}^{k}(\sigma_{m}^{k}-\sigma_{t})}E_{0}$$

Substituting these expressions into the overall current density equation yields the following:

(21) $$\sum_{i=x,\;y,\;z}\left.\frac{V_{CB}}{\alpha_{CB}}\;\;\frac{\sigma_{t}-\sigma_{CB}^{i}}{\sigma_{t}+B_{CB}^{i}\left(\sigma_{CB}^{i}-\sigma_{t}\right)}\right.+\sum_{k=X,Y,Z}\left.\left(1-\frac{V_{CB}}{\alpha_{CB}}\right)\frac{\sigma_{t}-\sigma_{m}^{k}}{\sigma_{t}+B_{m}^{k}\left(\sigma_{m}^{k}-\sigma_{t}\right)}\right.=0$$

where $V_{CB}$ is the volume fraction of CB nanoparticles, and $\alpha_{CB}={\left(\frac{R}{R+t}\right)}^{3}$ accounts for the ratio of the CB core to the effective nanoparticle volume. The depolarization factors $B_{CB}$ and $B_{m}$ are equal to 1/3 for the spherical particles.

Finally, the overall electrical conductivity of the CB/polymer nanocomposite is obtained from the following equation:

(22) $$\left[\left.\left.\frac{V_{CB}}{3\alpha_{CB}}\right.\left[\frac{\sigma_{t}-\sigma_{CB}}{\sigma_{t}+B_{CB}\left(\sigma_{CB}-\sigma_{t}\right)}+\frac{4\left(\sigma_{t}-\sigma_{CB}\right)}{2\sigma_{t}+\left(1-B_{CB}\right)\left(\sigma_{CB}-\sigma_{t}\right)}\right]+\right.3\left(1-\frac{V_{CB}}{\alpha_{CB}}\right)\left[\frac{\sigma_{t}-\sigma_{m}}{2\sigma_{t}+\sigma_{m}}\right]\right]=0$$

In this model, the electrical conductivity of the interphase region can be further expressed as $\sigma_{int}=\frac{d_{c}}{A_{c}R_{int}}$, where $d_{c}$ is the tunneling distance, $R_{c}$ is the contact area, and $R_{int}$ is the tunneling resistance as reported in [10,16,25].

(23) $$R_{int}(d_{c})=\left.\frac{d_{c}h^{2}}{A_{c}e^{2}\left.\sqrt{2m\lambda_{l}}\right.}\exp\left(\frac{4\pi d_{c}\left.\sqrt{2m\lambda_{l}}\right.}{h}\right)\right.$$

where h, e, m, and $\lambda_{l}$ are the Planck constant, electron charge, electron mass, and potential barrier height, sequentially. This relation shows the impact of tunneling distance and potential barrier height on the probability of electron transport across the interphase.

### 2.2. Effective Electrical Conductivity of SiC/CB Hybrid Composites

Dispersing CB nanoparticles into a non-conductive polymer such as epoxy transforms the polymer into a conductive matrix. This modification enhances the general electrical properties of the material, specifically improving the electrical conductivity of random SiC composites. The electrical conductivity of the new matrix is computed using Equation (22). Herein, a closed-form model designed to evaluate the electrical conductivity of the ternary CB/SiC/polymer composite is presented. Assume an arbitrarily dispersion of SiC whiskers within the conductive nanocomposite matrix. When the volume content of SiC surpasses a certain critical threshold, the electrical conductivity of SiC composites increases significantly. The percolation threshold $\left(V_{c}\right)$ assuming a random distribution of SiC whiskers, can be determined using the following expression [15,16,26]:

(24) $$V_{c}\left(H\right)=\frac{9H(1-H)}{2+15H-9H^{2}}$$

where H is obtained from the following equation:

(25) $$H\left(AR\right)=\frac{1}{{AR}^{2}-1}\left[\frac{AR}{\sqrt{{AR}^{2}-1}}\ln\left(AR+\sqrt{{AR}^{2}-1}\right)-1\right]$$

where AR represents the aspect ratio of SiC, defined as AR=l/D, and l is the length and D is the diameter of the SiC whisker. The percolation threshold represents the critical volume fraction of SiC at which there is a noticeable and rapid increase in the electrical conductivity.

The electrical conductivity of a microfiber-filled composite can be described by the following equation [2,15]:

(26) $$\frac{\sigma_{c}}{\sigma_{m.con}}=1+\frac{V_{SiC}}{3}\left[\xi \frac{\sigma_{SiC}}{\sigma_{m.con}}+\left(1-\xi \right)\frac{1}{\frac{\sigma_{m.con}}{\sigma_{SiC}}+H}\right]$$

where $\sigma_{m.con}$ is the electrical conductivity of the conductive matrix. Additionally, $\sigma_{SiC}$ and $V_{SiC}$ represent the electrical conductivity and the volume fraction of SiC, respectively. The variable ξ is the fraction of percolated SiC whiskers, and it can be roughly calculated as follows [15,27]:

(27) $$\xi =\frac{V_{SiC}^{1/3}-V_{c}^{1/3}}{1-V_{c}^{1/3}}$$

It is worth mentioning that $V_{SiC}$ ranges from $V_{C}$ to 1, and the value of ξ varies from 0 to 1.

## 3. Results and Discussion

### 3.1. Validation

In this section, the proposed modeling approach is validated by comparing its predictions with the available results from the literature. Two sets of validation are performed: first, the electrical conductivity of CB-reinforced polyurethane (PUR) nanocomposites is compared with experimental data from the study by Rebeque et al. [28]; second, the electrical conductivity of short carbon fiber (SCF) reinforced polymer-based composites is validated against the results provided by Pal and Kumar [29].

**Figure 2:**
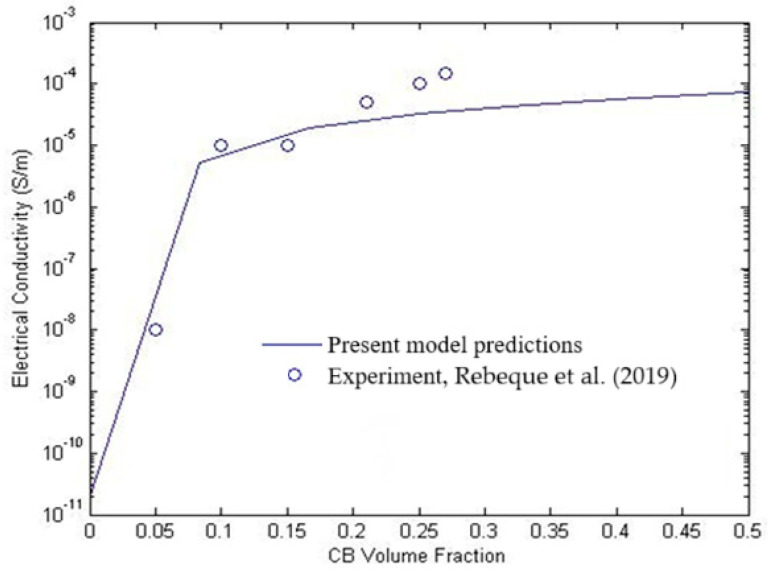
Comparison between the present model predictions and the experimental results obtained from the CB/PUR nanocomposite specimen [28].

First, the experimental data related to CB/PUR nanocomposites is scrutinized to validate the developed formulation herein [28]. For the sake of comparison, the Planck constant h, the electron charge e, electron mass m, and potential barrier height $\lambda_{l}$ are considered as $6.626068\times 10^{-34}\;m^{2}kg/s$, $-1.602176565\times 10^{-19}C$, $9.10938291\times 10^{-31}\;kg$, and 1 eV, in sequence. The specifications of the CB, the interphase region, and the matrix are as follows: radius of the CB R=17.5 nm, thickness of the interphase t=15 nm, conductivity of the CB and PUR $\sigma_{CB}=200\;S/m$ and $\sigma_{m}=1.87\times 10^{-11}S/m$, respectively, and the tunneling distance $d_{c}=2\;nm$. The model’s predictions are compared with the experimental data for different CB volume fractions to assess its accuracy in capturing the percolation behavior and effective electrical conductivity. Figure 2 presents the comparison between the predicted and experimental values of electrical conductivity for the CB/PUR nanocomposites. The model accurately captures the rapid increase in conductivity as the CB volume fraction exceeds the percolation threshold, showing good agreement with experimental data [28].

**Figure 3:**
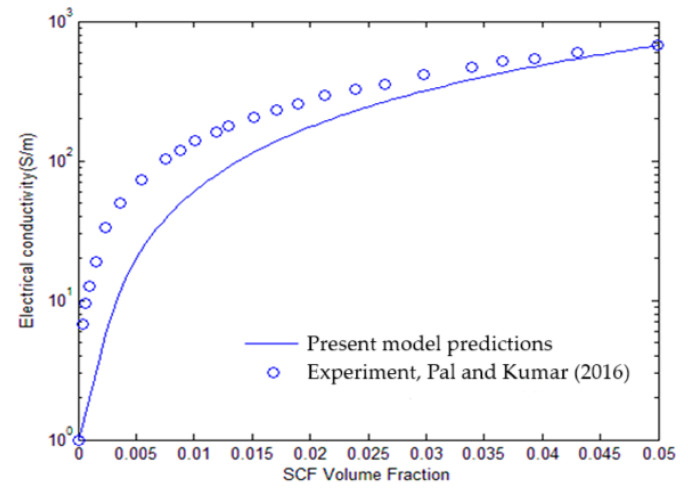
Comparison between the present model predictions and the results of Pal and Kumar for the SCF/polymer-based composite specimen [29].

The second validation is performed using the results of the electrical conductivity for the SCF-reinforced polymer-based composites from Pal and Kumar [29]. The results provide the relationship between composite electrical conductivity and SCF volume fraction dispersed into the matrix. The electrical conductivities of SCF ($\sigma_{SCF}$) and the matrix $\sigma_{m}$, and the fiber aspect ratio AR are considered as 66,250 S/m, 1 S/m, and 100, respectively. Present predictions of the electrical conductivity for the SCF-reinforced composites are compared with the results reported in Ref. [29], and depicted in Figure 3. The comparison indicates that the model successfully predicts the electrical conductivity behavior over a range of SCF volume fractions, with a good alignment between the two sets of results across the entire range. This suggests that the model can accurately represent the conductive network formation within SCF/polymer composite systems, taking into account the aspect ratio and volume fraction of the SCF reinforcements.

### 3.2. Parametric Studies

In this section, a comprehensive investigation on the multiscale modeling of ternary CB/SiC/polymer hybrid composites is presented. To this end, a detailed parametric study is conducted on the electrical conductivity of CB/SiC whisker-reinforced polymer composites. The analysis focuses on the effect of several key parameters, such as the volume fraction of CB and SiC, CB nanoparticle size, interphase thickness, SiC whisker conductivity, and aspect ratio on the effective electrical conductivity of the composites. The specifications of the polymer matrix, CB, and SiC whiskers are provided in Table 1 [15,16,29,30,31]. These values are used as the base parameters for the simulation, and any variations or adjustments to these parameters will be explicitly mentioned in the corresponding figure.

**Figure 4:**
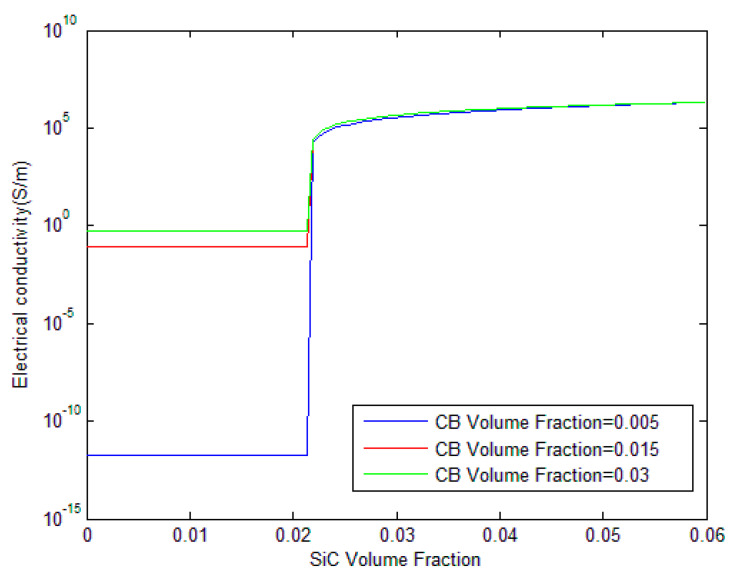
Influence of CB volume fraction on the electrical conductivity of CB/SiC/polymer hybrid composites.

In Figure 4, the influence of varying CB volume fractions on the electrical conductivity of CB/SiC/polymer composites is depicted. Three different volume fractions of CB are compared: 0.005, 0.015, and 0.03. The figure shows that, before reaching the percolation threshold, there is a significant difference in the electrical conductivity between the curves. The lowest CB volume fraction (0.005, represented by the blue line) yields the least conductivity, indicating the scarcity of conductive pathways within the composite. In contrast, the composites with 0.015 and 0.03 CB volume fractions, show higher conductivity even before the percolation threshold is reached. This is due to the increased likelihood of conductive network formation as the CB content rises. Thus, the formation of more conductive networks can be an important parameter that governs the electrical conductivity of the hybrid composites before percolation. After the percolation threshold; however, all curves converge, demonstrating that beyond this point, the volume fraction of CB has a little additional impact on conductivity, and the system achieves a saturated level of conductivity.

In the present study, a uniform dispersion of nanoparticles without any aggregation is considered in the polymer matrix. Also, it is noticed that the model assumes uniform tunneling distances without considering potential variations due to particle aggregation, which could impact the conductivity predictions. It has been reported that uniform dispersion without any aggregation of nanoparticles may be desirable to improve the electrical properties of composites [23,32,33]. But, other experimental works indicate that a less than uniform dispersion with carbon-based nanofiller aggregations may favor the formation of conductive networks in the composite materials [23,34,35].

**Figure 5:**
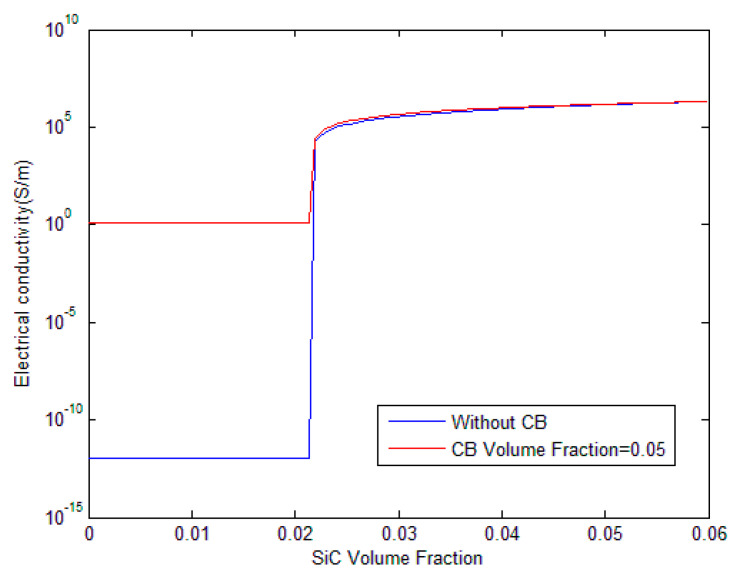
Influence of CB dispersion on the electrical conductivity of CB/SiC/polymer hybrid composites.

Figure 5 demonstrates the effect of CB dispersion into the polymer matrix on the electrical conductivity of the hybrid composites. Two cases are presented: one without any CB (blue line) and one with a CB volume fraction of 0.05 (red line). The figure clearly shows that the presence of CB drastically enhances the electrical conductivity of the hybrid composites. The blue line, representing the absence of CB, shows a low baseline conductivity across the range of SiC whisker volume fractions. In comparison, the red line with 0.05 CB exhibits significantly higher conductivity throughout the entire range. Interestingly, both curves exhibit the same percolation threshold, indicating that while the addition of CB improves the overall conductivity, it does not shift the threshold.

**Figure 6:**
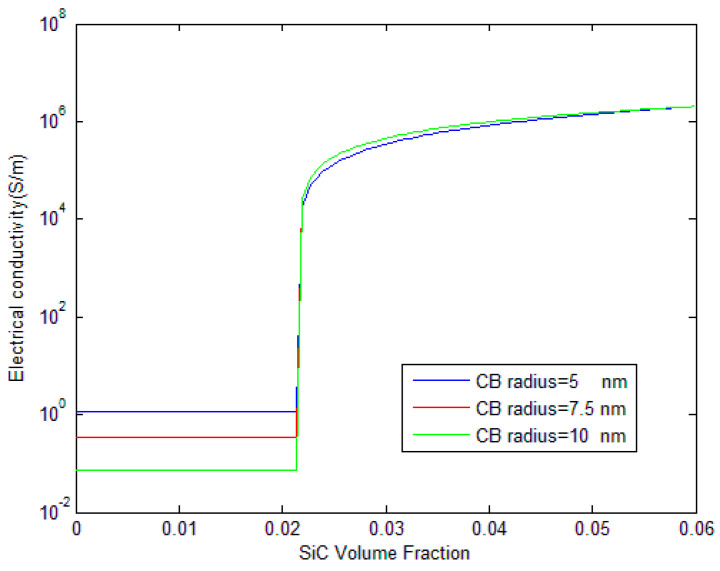
Influence of CB radius on the electrical conductivity of CB/SiC/polymer hybrid composites.

In Figure 6, the influence of CB nanoparticle radius on the electrical conductivity is examined. Three radii are considered: 5 nm, 7.5 nm, and 10 nm. The results indicate that smaller CB particles (blue line, 5 nm) provide significantly higher conductivity before the percolation threshold compared to larger particles. This may be attributed to the greater number and larger surface area of smaller particles, which increases the probability of forming conductive paths via tunneling mechanisms. The figure shows that the blue line exhibits the highest conductivity before percolation, followed by the red and green lines for 7.5 nm and 10 nm CB radii, sequentially. After percolation, the differences between the curves are less pronounced, though the green line for 10 nm shows a slight increase in conductivity compared to the others. It has been shown that small size functionalized nanoparticles can increase the conductivity drastically and improve the overall physical properties of composites [36,37,38,39,40,41,42,43,44]. Yaduvanshi et al. observed that DC conductivity of HAT4 has enhanced by two orders of magnitude due to copper nano-particles [42]. Therefore, a reason for the enhancement of the electrical conductivity before the percolation threshold may be described by the existence of more conductive networks for nanoparticles with smaller size for the same percentage.

**Figure 7:**
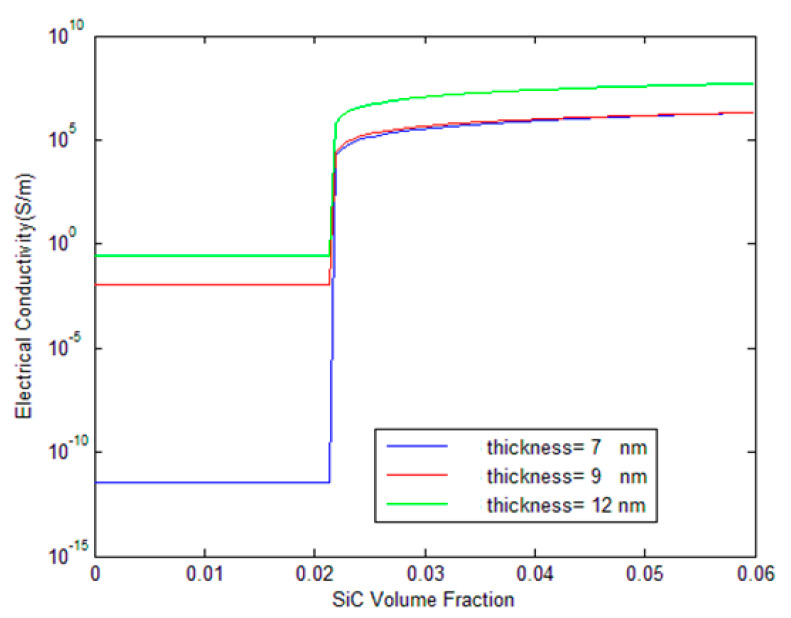
Influence of CB interphase thickness on the electrical conductivity of CB/SiC/polymer hybrid composites.

During the micromechanical modeling of carbon nanoparticle-polymer nanocomposites, an interphase is captured among the nanofiller and the polymer matrix which characterizes the non-bonded van der Waals interaction between them. Such an interphase can be regarded as an equivalent solid continuum with a definite thickness [45,46,47,48]. The effect of CB interphase thickness on the effective electrical conductivity of polymer composites containing SiC whisker/CB hybrids is illustrated in Figure 7. Three interphase thicknesses (7 nm, 9 nm, and 12 nm) are compared, and the results show that thicker interphases lead to higher conductivity before and after the percolation threshold. The thinnest interphase (7 nm) shows the lowest conductivity before percolation, while the thickest interphase (12 nm) results in the highest conductivity across the entire range. This is because a thicker interphase provides a larger region for conductive electron transport, enhancing the overall conductivity of the composite. After the percolation threshold, the green line continues to exhibit higher conductivity than the others, demonstrating the continued benefit of a thicker interphase in facilitating electron transport.

**Figure 8:**
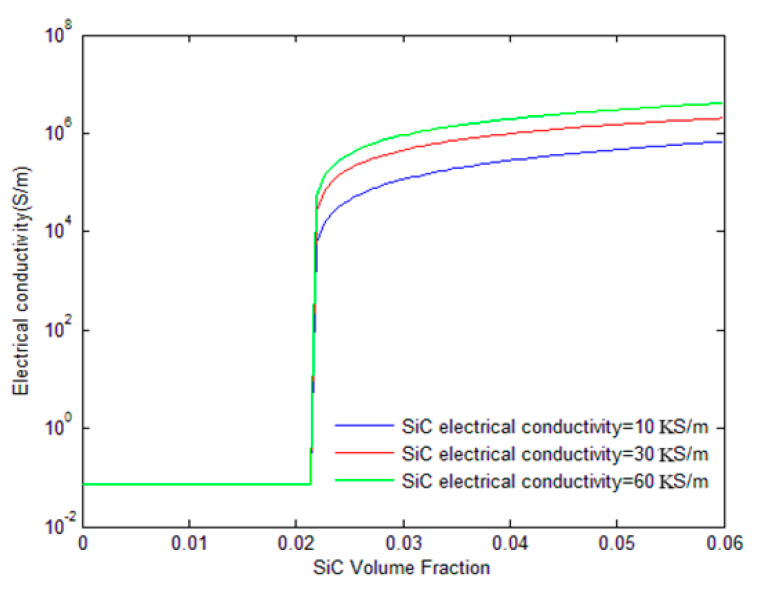
Influence of SiC whisker’s conductivity on the electrical conductivity of CB/SiC/polymer hybrid composites.

Figure 8 explores the influence of SiC whisker conductivity on the overall electrical conductivity of the hybrid composites. Three different SiC conductivities are considered: 10 × 10^3^ S/m, 30 × 10^3^ S/m, and 60 × 10^3^ S/m. The figure shows that there is little difference in conductivity between the three cases before the percolation threshold, as the SiC whiskers do not significantly contribute to the conductive network in this region. However, after the percolation threshold, the green line (60 × 10^3^ S/m SiC conductivity) demonstrates the highest conductivity, followed by the red line (30 × 10^3^ S/m), and the blue line (10 × 10^3^ S/m). This suggests that higher SiC conductivities enhance the capability of conductive pathways, thereby improving the overall electrical performance of the composite.

**Figure 9:**
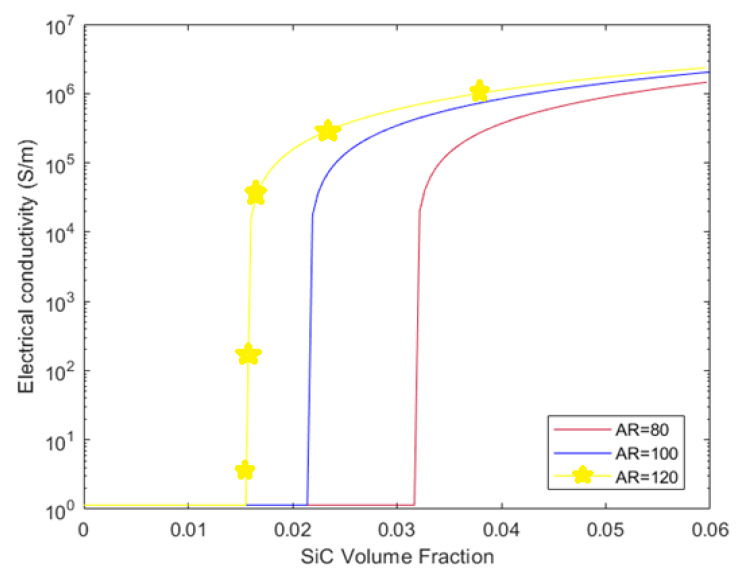
Influence of SiC whisker’s aspect ratio on the electrical conductivity and the percolation threshold of CB/SiC/polymer hybrid composites.

Figure 9 presents the effect of the SiC whisker aspect ratio on the electrical conductivity of the polymer composites containing micro/nano hybrids. Three aspect ratios are compared: 80, 100, and 120. The results indicate that the aspect ratio has a significant impact on both the percolation threshold and the post-percolation conductivity. The yellow line, representing the highest aspect ratio (120), reaches the percolation threshold at a lower SiC volume fraction compared to the red and blue lines, indicating that higher aspect ratios lead to earlier percolation. This is because longer whiskers are more efficient at forming conductive networks at lower volume fractions. After percolation, the yellow line remains higher than the others, showing that a higher aspect ratio not only reduces the percolation threshold but also enhances conductivity post-percolation. The outcomes offer that the electrical conductivity of hybrid composites is improved by the increase in the SiC whisker aspect ratio. Thus, fabricating a hybrid composite with a high-length whisker can be more effective in its electrical conductivity.

## 4. Conclusions

In this study, an analytical micromechanics method was developed to investigate the effective electrical conductivity of hybrid polymer composites reinforced with SiC whiskers and CB nanoparticles. A two-phase modeling approach was employed, wherein CB nanoparticles were first dispersed in a non-conductive polymer matrix to form a conductive nanocomposite, followed by the incorporation of randomly oriented and dispersed SiC whiskers as a secondary reinforcement to further enhance the composite’s electrical performance. The model captured critical microstructural parameters such as the volume fraction and size of CB nanoparticles, as well as the aspect ratio and volume fraction of SiC whiskers. The model predictions were validated using available results from the literature for different types of composite systems, demonstrating a good agreement between the two sets of results. The findings revealed that increasing the volume fraction of CB significantly enhances electrical conductivity before the percolation threshold, while the influence diminishes after percolation is achieved. Furthermore, the radius and interphase thickness of CB nanoparticles were shown to have a critical impact on the composite’s conductivity, with smaller radii and thicker interphase regions contributing to the improved electrical conductivity of the CB/SiC/polymer composites. The study also highlights the role of SiC whisker aspect ratio, with higher aspect ratios resulting in significantly lower percolation thresholds and higher electrical conductivity in the post-percolation regime. This work provides a robust framework for understanding and predicting the electrical conductivity of SiC/CB hybrid polymer composites. The results indicate that optimizing the microstructural characteristics of both CB nanoparticles and SiC whiskers is essential for enhancing the electrical performance of the composite.

## Figures and Tables

**Table 1:** Specifications of the matrix, CB and SiC fillers.

CB	R=5 nm	$\sigma_{CB}=10^{4}\frac{S}{m}$	$\sigma_{m}=10^{-12}\frac{S}{m}$	$\lambda_{l}=1.1\;eV$	$d_{c}=1\;nm$	t=10 nm
SiC	$D_{SiC}=2\;\mu m$	$l_{SiC}=200\;\mu m$	$\sigma_{SiC}=3\times 10^{4}\frac{S}{m}$	AR=100	-	-

## Data Availability

Could be made available on request.
